# Genome sequence of a Florida Clade 1 case of equine influenza in Switzerland

**DOI:** 10.1128/mra.00635-26

**Published:** 2026-07-16

**Authors:** Jakub Kubacki, Sandra Renzullo, Natalie Miller-Collmann, Dilara Lale, Claudia Bachofen, Michele Wyler

**Affiliations:** 1 Institute of Virology and Immunology 87699, Mittelhäusern, Switzerland; 2 Department of Infectious Diseases and Pathobiology, Vetsuisse Faculty, University of Bern 27210, Bern, Switzerland; 3 Equine Department, Vetsuisse Faculty, University of Zurich 27217, Zurich, Switzerland; Katholieke Universiteit Leuven, Leuven, Belgium

**Keywords:** equine influenza, genome, influenza A virus

## Abstract

Here, we report the whole-genome sequence of A/*Equus caballus*/Zuerich/260760/2026, an equine influenza virus (H3N8) belonging to Florida Clade 1, detected in Switzerland following increased reports of equine influenza in several European countries since 2025.

## ANNOUNCEMENT

Equine influenza (EI) is a highly transmissible acute respiratory disease of horses, causing fever, coughing, and reduced performance. Secondary infections may lead to bronchitis and/or bronchopneumonia (1). The causative agents are Alphainfluenzavirus from the *Orthomyxoviridae* family, notably of the H3N8 subtype. Today, major outbreaks are mostly caused by two sublineages originating from the American lineage, that is, Florida Clade 1 (FC1) and Florida (FC 2) (2).

EI is not an officially notifiable disease in Switzerland. Prevention strongly relies on voluntary reporting and vaccination (compulsory in horses competing in equestrian sports). Due to the possibility of genetic shift and drift, surveillance efforts are pivotal in guaranteeing antigenically relevant vaccines.

In February 2026, the first case of EI in over 10 years was reported in Switzerland (https://www.equinella.ch/home/, accessed 15 May 2026). This is in line with the apparent emergence of EI in various European countries since 2025 (https://equinesurveillance.org, accessed 15 May 2026). Since the initial detection, Switzerland registered further two cases of EI, one of them sequenced in this study.

A 3-year-old, non-vaccinated Pura Raza Española gelding was presented on 21st April with cough and fever 3 days after being imported from Spain. The initial ultrasound examination revealed pulmonary consolidation. Together with the travel history, these findings led to a presumptive diagnosis of shipping fever. To rule out a viral infection, a nasal swab was collected and tested positive for EI virus with a Ct-value of 23.4 using reverse transcription quantitative polymerase chain reaction (3).

RNA was extracted from nasal swab using NucleoMagVET Extraction Kit (Macherey-Nagel, Germany). Complementary DNA conversion and amplification were performed using the Optimized ONT Protocol by Goraichuk et al. (4). PCR amplicons were cleaned up using the NucleoSpin Gel and PCR Clean-up Kit (Macherey-Nagel, Germany). Quantity of DNA was assessed using the Qubit dsDNA High Sensitivity assay (Thermo Fisher Scientific, USA). The amplicons were prepared using Rapid Sequencing Kit V14 (Oxford Nanopore Technologies, UK) and subsequently sequenced on an Oxford Nanopore platform (MinION Mk1C) with a R10.4.1 flow cell. The basecalling was performed using the Dorado fast model (Oxford Nanopore Technologies, UK).

A total of 142,206 reads passed basecalling (*N*50 = 924). The reads were *de novo* assembled using IRMA (v1.2.0, https://wonder.cdc.gov/amd/flu/irma/) with the FLU-minion module generating eight full-length influenza virus sequences. Overall sequencing depth was 4,736×, ranging from 1,148× to 7,708× for nucleocapsid protein (NP) and hemagglutinin (HA), respectively. Quality control and annotation were performed through the automated GenBank submission portal pipeline for influenza virus.

The HA segment of A/*Equus caballus*/Zuerich/260760/2026 could be assigned to FC1 (99.65% identity, Table 1), the sublineage historically associated with EI cases in North America. Thus, our results are in line with observations from recent outbreaks that suggest a weakening of a clear relationship between geography and sublineage occurrence (5, 6). Likewise, the remaining segments showed high similarity to the strain A/Percheron Horse/NY/25-024601-002-original/2025 detected in 2025 in New York (Table 1), as well as a recently published strain from Italy (A/equine/Puglia/MT944_2/2026, only non-polymerase segments).

**TABLE 1:** Genome properties A/*Equus caballus*/Zuerich/260760/2026^*a*^

Protein	Length (nt)	GC content (%)	Number of reads	GenBank ID	Genomic similarity (%)	Nearest genomic match
Polymerase PB2	2,280	42.54	20,211	PZ397321	99.78	PX579503.1
Polymerase PB1	2,274	41.78	13,374	PZ397328	99.87	PX579504.1
Polymerase (PA)	2,151	40.59	14,405	PZ397325	99.86	PX579505.1
Hemagglutinin (HA)	1,695	40.00	32,191	PZ397322	99.65	PZ381563.1
Nucleocapsid protein (NP)	1,497	45.22	2,885	PZ397324	99.93	PZ381567.1
Neuraminidase (NA)	1,413	39.99	5,367	PZ397323	99.86	PZ381564.1
Matrix protein MP	982	47.35	11,214	PZ397327	100	PX579493.1
Nuclear export protein (NS)	838	41.17	19,150	PZ397326	99.76	PX579510.1
Genome	13,130	42.06	118,797			

^*a*^ nt, nucleotide.

## Data Availability

The complete genome was deposited in the National Center for Biotechnology Information GenBank under accession numbers PZ397321–PZ397328 and reads on European Nucleotide Archive PRJEB113284.
